# Noninvasive prenatal testing as compared to chorionic villus sampling is more sensitive for the detection of confined placental mosaicism involving the cytotrophoblast

**DOI:** 10.1002/pd.5766

**Published:** 2020-06-29

**Authors:** Diane Van Opstal, Geerke M. Eggenhuizen, Marieke Joosten, Karin Diderich, Lutgarde Govaerts, Robert‐Jan Galjaard, Attie Go, Maarten Knapen, Marjan Boter, Wai Y. Cheung, Nicole van Koetsveld, Stefanie van Veen, Walter G. de Valk, Fernanda Jehee, Femke de Vries, Iris Hollink, Lies Hoefsloot, Malgorzata Srebniak

**Affiliations:** ^1^ Department of Clinical Genetics Erasmus Medical Center Rotterdam The Netherlands; ^2^ Department of Obstetrics and Fetal Medicine Erasmus Medical Center Rotterdam The Netherlands

1

**What's already known about this topic?**

- Confined placental mosaicism (CPM) can prenatally be detected with chorionic villus sampling (CVS) and noninvasive prenatal testing (NIPT).
- Chromosomally abnormal cells may be restricted to a small part of the placenta.
- The level of mosaicism detected by CVS does not always reflect the level present in the term placenta.

**What does this study add?**

- NIPT as compared to CVS is more sensitive for detection of CPM involving the cytotrophoblast that is restricted to a (small) part of the placenta.

Confined placental mosaicism (CPM) is defined as a chromosomally abnormal cell line restricted to the placenta, while the fetus is chromosomally normal. It was first described in 1983 in term placentae. CPM can prenatally be detected by chorionic villus sampling (CVS) or by noninvasive prenatal testing (NIPT) using cell‐free (cf) DNA. NIPT investigates DNA released from the cytotrophoblast (CTB) in maternal blood plasma. CPM is now recognized as the major origin of discordant NIPT results.

Little is known about the sensitivity of NIPT for detection of CPM. Brison et al (2018) found evidence that NIPT is more sensitive for the detection of placental mosaicism due to the observation of a higher proportion of mosaicism for the common aneuploidies with NIPT as compared to conventional karyotyping.^1^ In contrast, Benn et al^2^ showed a significantly lower sensitivity for the detection of rare autosomal aneuploidies (RATs), mostly involved in CPM, for NIPT (0.32%) vs CVS (0.41%). The use of study cohorts with probably different a priori risk figures for CPM, may explain the conflicting results of both studies. Moreover, whereas placental studies shed some light on the correlation between cytogenetic results of CVS and those from term placentae, little is known about how cytogenetic results of NIPT relate to those from CVS and placenta. Papers on placental cytogenetic studies after NIPT are rare and amniocentesis is generally the preferred technique for confirmatory diagnostic testing after an abnormal NIPT result. Potential detection of chromosomal mosaicism in CV, which may require an undesired second invasive procedure for clarification of the fetal karyotype, may discredit CVS. However, if both cell layers of CV (CTB and mesenchymal core [MC]) are investigated separately, thus enabling differentiation between their respective chromosomal constitution, the risk of a confirmatory amniocentesis after CVS is predicted to be low for the common trisomies (eg, trisomy 21 (2%), trisomy 18 (4%), and trisomy 13 (8%‐22%)^3^, ^4^). On the contrary, if NIPT indicates another trisomy, CPM is the most likely reason for this result. In such cases CVS for confirmation is only recommended for RATs that are mostly involved in CPM type 1, like trisomy 3, 7, 8, 9, 20.^3^ In all other cases, amniocentesis is indeed the preferred confirmatory test. Hence little is known about the representation of the placental chromosomal constitution in the cfDNA fraction in cases of CPM and about the sensitivity of NIPT to detect it. It is assumed that the entire placental trophoblast sheds cfDNA into the maternal circulation and that a CPM restricted to smaller placental areas may be detected by NIPT and missed by CVS.^1^ However, as far as we know, there are no studies comparing NIPT, CVS and placenta cytogenetic data. Based on four cases with normal CVS results after genome‐wide (gw) NIPT revealed a RAT, we show evidence that NIPT is better able to detect (low‐level) placental mosaicism involving the cytotrophoblast than CVS.

Genome‐wide NIPT was performed as part of the Dutch Trident 2 study (Trident = Trial by Dutch laboratories for Evaluation of NIPT), using shallow massively parallel sequencing and WISECONDOR for analysis.^5^ The four cases presented here involved one case of trisomy 5 and trisomy 7 and three cases of trisomy 8. According to our local protocol, a CVS was recommended, which was performed transabdominally in all cases. Cytogenetic investigations of first trimester CV were performed with SNP array (Illumina Infinium GSA + MD‐24 v1.0 BeadChip genotyping array) on DNA isolated from the CTB and MC that were separated as described previously.^6^ Maternal genomic DNA was investigated as well to exclude a maternal origin of the chromosomal aberration. In all four cases, a normal result was achieved in CV (both CTB and MC) and maternal blood. The test characteristics of NIPT (gestational age (GA), fetal fraction (FF) (SeqFF)^7^ and z‐score (chromosome‐wide aneuploidy test [CWAT]^8^) and CVS (GA and amount of CV) are shown in Table 1. Since maternal genomic DNA was normal in all cases, a diagnosis of CPM was most likely, despite normal CV results. After birth, we collected the placentae and performed cytogenetic analysis of four CV biopsies from four quadrants, with methods described for first trimester CV (Table 1). In all cases, the chromosomal aberration was confirmed in the term placenta. In two cases, it was present only in one of four biopsies, involving a 100% trisomy 5 and trisomy 7 in case 1 (Figure 1), but a very low level mosaic in case 2. The presence of only 10% abnormal cells in one biopsy in case 2 was sufficient to lead to an abnormal NIPT‐result. However, sampling of only 4 × 1 cm^3^ biopsies does not exclude higher levels of trisomic cells elsewhere in the placenta. In cases 3 and 4, a 100% trisomy was present in two of the four biopsies, while first trimester CV showed normal results, confirming the nonrepresentativity of first trimester CV for the placenta as a whole, as illustrated in the past. Our study shows a higher sensitivity of NIPT for detection of CPM involving the cytotrophoblast as compared to CVS.

**TABLE 1:** Four cases of confined placental mosaicism detected with gwNIPT and confirmed in placenta but with normal results in first trimester CVS: cytogenetic results with SNP array during pregnancy in CV and blood of pregnant woman, and after birth in placenta and cord blood as well as clinical outcome

Case	NIPT result	GA	FF	z‐score (CWAT)				Postnatal cytogenetics									Clinical outcome
								Placenta (4 CV biopsies)									
					cytogenetics in CVS (normal CTB and MC)			% trisomy in CTB 1‐4				% trisomy in MC 1‐4					
					GA^a^	mg CV	Maternal blood	1	2	3	4	1	2	3	4	Cord blood	
1	T5 and T7	12	10.4	13.5 (chr5) 11.6 (chr7)	14 3/7	8	N	0	0	100 (T5) 100 (T7)	0	0	0	0	0	0	Spontaneous labor at 39 6/7, 2888 g, p10.3 No congenital malformations Uneventful pregnancy
2	T8	12	7.1	7.4	14 1/7	15	N	0	0	10	0	0	0	0	0	‐	Spontaneous labor at 39 3/7, 4062 g, p94 No congenital malformations Uneventful pregnancy.
3	T8	17	7.5	9.5	19 1/7	20	N	20	100	0	100	0	10	0	0	‐	Premature delivery at 20 1/7 wks
4	T8	11 5/7	11.6	32.6	14 4/7	40	N	100	100	0	0	0	0	0	0	0	Spontaneous labor at 39 1/7, 3448, p42 No congenital malformations Uneventful pregnancy

^a^ Note that CVS took place in the second trimester (between 14 and 20 weeks) due to late NIPT (between 11 and 17 weeks). ‐, Not performed; CTB 1–4, cytotrofoblast of 4 CV biopsies; CV(S), chorionic villi (sampling); CWAT, z‐score of chromosome wide aneuploidy test in WISECONDOR; FF‐fetal fraction by SeqFF, fetal cell‐free DNA fraction using sequence reads counts^7^; GA, gestational age in weeks; MC 1–4, mesenchymal core of 4 CV biopsies; mg, miligram; p, percentile; wks, weeks of gestation; T, trisomy.

**FIGURE 1:**
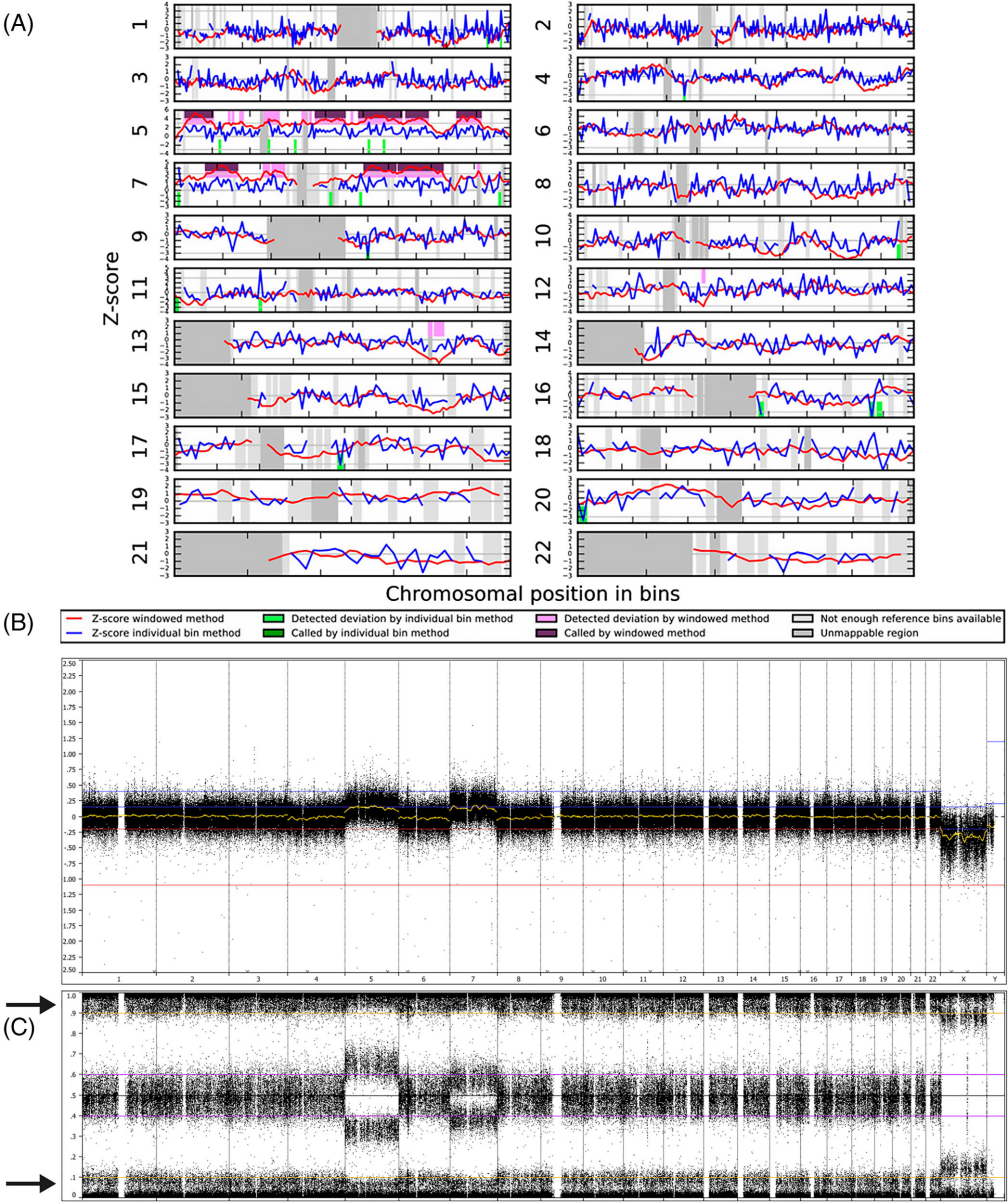
NIPT and array results in case 1. A, WISECONDOR plot showing the abnormal NIPT result in case 1 with a trisomy of both chromosomes 5 and 7. B and C, array result of the cytotrophoblast of placental biopsy 3. B, shows the whole genome LogR and C, the whole genome B‐allele frequency (BAF). Both reveal a nonmosaic trisomy 5 and trisomy 7 in the presence of approximately 10% maternal cell contamination. The latter can be seen in the BAF profile at a BAF of 0 and 1.0 (arrows). The differences in the BAF‐profiles of chromosomes 5 and 7 are caused by a different parental origin of both trisomies, with trisomy 5 having a maternal and trisomy 7 a paternal origin [Colour figure can be viewed at wileyonlinelibrary.com]

A striking observation is that whereas the results in placental studies of cases 3 and 4 were comparable, z‐scores of the NIPT were much higher in case 4 (32.6) as compared to case 3 (9.5). This may be partly due to a higher FF (11.6% in case 4 vs 7.5% in case 3), however, other factors may be involved that can explain this difference. Firstly, only 4 biopsies of 1 cm^3^ were investigated leaving the largest part of the placenta uninvestigated, which may contain much higher levels of trisomic cells in case 4 as compared to case 3. Secondly, it is also possible that apoptotic activity in the affected placental parts in case 4 is much higher than in case 3, leading to a higher trisomic cfDNA fraction. In order to get more insight into the representativeness of NIPT for the placenta anomaly, further studies are necessary and preferably should involve more than four placental biopsies.

In three of the four cases, all involving CPM type 1 with the chromosome aberration restricted to the CTB, children without congenital anomalies and with appropriate birth weights were born, as can be expected for this CPM type. Clinical outcome data are shown in Table 1. In case 3 with the chromosome aberration present in both cell layers, a premature delivery due to a rupture of membranes occurred at 20 1/7 weeks. Unfortunately, no cord blood could be obtained and therefore, a fetal trisomy 8 could not be excluded.

CPM is associated with an increased risk for preterm birth, small for gestational age newborns, and adverse pregnancy outcomes.^9^ This association especially exists for CPM type 3, mostly of meiotic origin, in which both CTB and MC of first trimester CV are affected, often with high percentages of abnormal cells, and less for CPM type 1 (only CTB affected) and type 2 (only MC affected).^9^ Discrimination of the various types of CPM is only possible when both the CTB and MC of CV are investigated. When both cell layers are affected with high levels of abnormal cells, while the fetus is chromosomally normal, proper clinical follow‐up investigations like expert ultrasound can be recommended. However, if potential CPM is detected with NIPT, no differentiation between CPM type 1 and 3 is possible since only the CTB is investigated with NIPT. Also, little is known about the extent of the distribution of abnormal cells over the placenta when NIPT reveals CPM. Moreover, as shown in this paper, NIPT seems to be very sensitive for detection of CPM, even if restricted to a small area of the placenta, with probably less clinical consequences. This all complicates predictions on the clinical relevance of CPM when detected with NIPT. Further research is necessary in order to learn to differentiate clinically relevant CPM from benign CPM. Recently, Pertile et al (2017) and Brison et al (2018) found an association between trisomic fraction and pregnancy outcome. When a trisomic fraction as compared to fetal fraction was low, pregnancy outcome was favorable and if trisomic fraction was high there was an increased risk for adverse outcome such as miscarriage, intrauterine fetal death, intrauterine growth retardation^1^, ^10^ This shows that the trisomic fraction may be a good indicator for aneuploidy‐load in the placenta, and its calculation probably may improve clinical guidance of the pregnancy.^1^

In conclusion, the present study shows that NIPT seems to be more sensitive than CVS for the detection of CPM involving the cytotrophoblast. However, the ability of NIPT to detect a low level mosaic restricted to a small placental part will probably be dependent on the FF. This study also gives more insight into the representation of CPM in the cfDNA fraction of maternal blood. However, more studies are necessary to understand the correlation between NIPT z‐scores/trisomic fraction and level and distribution of mosaicism in the placenta in order to learn to predict the clinical consequences of CPM, when detected with NIPT.

## 2 CONFLICT OF INTEREST

None declared.

## Data Availability

The data that support the findings of this study are available from the corresponding author upon reasonable request
